# Monthly Summary

**Published:** 1896-02

**Authors:** 


### Monthly Summary.

GOOD AND BAD TEETH.—That the tendency to decay of the teeth is in some way dependent upon a deficiency of lime salts has often been assumed; it is only lately however, that crucial investigations can be said to have settled the question. In a series of remarkably interesting articles, concluded in the current number of the *Journal of the British Dental Association*, on the Analysis of the Teeth, Mr. Charles Tomes records some quantitative estimates of his own and compares them with those of Dr. Black recently published in America. After describing in detail his method of estimating the various constituents of dentine, obtained from sets of teeth in his own posession, Mr. Tomes remarks: "Taking all teeth examined, the percentage of lime salts in dentine is 71.9. I am inclined, however, to think Dr. Black is right in thinking that the percentage of lime salts is not the factor which determines the liability to caries, for in one set of teeth, in which the wisdom teeth were barely in place, two bicuspids had been largely affected, there was incipient caries between many of the teeth one wisdom tooth was badly decayed and the incisors were markedly striated transversely; yet this set of teeth gave an average of 71.4 per cent. of salts, whilst another much worn dentition, quite free from any trace of caries, also gave 71.4 per cent. of salts; that is to say, both were 0.5 below the general average." It must certainly come as a surprise, not merely that teeth which we recognize as types as vigorous, healthy formation should be no better off in lime salts than those presenting all the signs of liability to decay, but, moreover, that teeth worn down by attrition, in which it has been assumed an extra calcification has taken place, should actually be below the averave percentage of lime salts. Another curious outcome of these investigations arose from a statement by Dr. Black that there were differences in the amount of mineral matter in teeth taken from the same individual. Mr. Tomes, in confirmation, adds: "But I also find an unexpected fact which is of much interest. The differences are not fortuitous, for I have found that the incisors and canines uniformly contained a smaller portion of mineral matter than the molars." He gives the proportion as follows: incisors, 71.2 per cent. inorganic salts and 28.8 per cent. organic matter and water; molars, 73 per cent. inorganic salts and 27 per cent. organic matter and water. This further appears to support the view that the proportion of lime salts is not a chief factor in the production of decay, for the molar teeth are certainly more prone to decay than the incisors, although according to these figures the former are richer in lime salts than the latter. Mr. Tomes has promised to carry his investigations further; meanwhile he suggests that "the difference between good and bad teeth may lie in the amount of combined water, in the proportion of calcium carbonate, in the nature of the organic matrix, or, finally it may not lie in the dentine at all, but may be a difference in the enamal."—*London Lancet.*

---

MAMMOTH TEETH UNEARTHED.—Mr. Nace Brock has shown two huge teeth which were dug out of a canal on Mr. Cad Koonce's land, near Richlands, Onslow county, N. C., at a depth of six feet. The teeth are adjudged to have belonged either to a mastodon or a megatherium, both of which animals are accounted by scientists to have existed ages before man, the former in highlands, the latter in lowlands. The mastodon was somewhat the larger of the two, but the megatherium had the greatest length. He was about thirty feet long, and by raising himself up and resting his front feet on tree limbs or other support he could graze upon the tops of moderate sized trees. It is to this latter animal that Prof. Charles Hallock, who has seen skeletons of each, supposes the teeth to have belonged.

Each tooth has two deep indentures on top in one direction, and a cross central one the other way. These give the top of the tooth somewhat the appearance of three rows of saw teeth with two teeth to the row.

They measure about 6 inches from the crown to the end of the roots, and they measure 6 inches across in one direction and four in the other. One tooth is nearly entire—only a small part of the roots having been broken off, the other is simply the upper half; the tooth that is entire weighs three pounds.

Some bones, seemingly remains of the animals to which the teeth belonged, were found also, but they were too decayed and crumbly to amount to much.

Mr. Hallock informs us that about fifty varieties of animal remains—some of extinct species and some of species still extant—can be found in the great South Carolina phosphate beds. The immense accumulations are accounted for by scientific men in this way. They say there were great floods which drowned out the animals, but the currents swept the great proportion of them to the places where the immense deposits are found, while here and there an animal or two would be caught and its remains left elsewhere, as in the case of this solitary animal now found in Onslow county.

Dr. Hallock gives us the following as to the origin of such deposits and the extinction of certain species:

"Historical geology might inform us that once upon a time, during the early part of the Cenozoic age, in the period termed tertiary, a mighty flood from which there was no escape submerged nearly the whole continent as it now sometimes does portions of the Mississippi Valley, and involved these innumerable creatures in one common destruction. Then did the overwhelming downpour of rain beat the helpless fowls to the earth.

"Here, in the still and stagnating waters, the processes of decomposition and sedimentary deposit went steadily on. The drift of other partial floods which succeeded gradually covered them. Silt, till, vegetable mould and all kinds of organic matter accumulated, including the decayed portions of the carcasses, and finally the lagoons filled up, the water evaporated or ran off, the land emerged or rather was raised by alluvial deposits carried from higher regions, plants and trees germinated, grew and maturated, and so by natural and intelligible processes the phosphate beds were formed. Portions of the bones and pachyderm did not decay, but became preserved or petrified. In the aggregate no better material for a compost heap could be gotten together.

These phosphate beds, therefore, are the records of the second great submergence of the continent, the first having taken place during the first glacial period, when there was no animal life. The partial submergences or lesser floods which occurred subsequently added to the depth of the deposits and to the assortment of animal remains, thus accounting for remains of extinct and extant creatures being found together. But it was during the great submergence that many of the now extinct animals disappeared."—*Forest and Stream.*

A CASE OF COMPLETE SEPARATION OF THE UPPER JAW.—A man, whose case Dr. G. H. Hopkins relates in the London lancet, November 2, aged 49 years, was admitted to Swansea Hospital on August 7, 1895. He had been struck on the back of the head by a wooden beam and knocked forward on to a coal truck, the sharp edge of which had caught him at the root of the nose. On examination the whole of the upper jaw was found to be detached from the skull, the nasal processes of the superior maxillary bones and the zygomatic processes of the malar bones being fractured. There was over an inch of separation in the middle line. The frontal sinus and anterior ethmoidal cells were opened up. The eyes were quite uninjured. The parts were cleaned and stitched up, free drainage being provided for by the nose. A Smith's gag was used, which kept the parts in very good position. The patient wore this continuously for a fortnight. He is, now quite well with the exception of slight ptosis of the right eye.

---

DEATH DUE TO DENTISTRY—A WOMAN'S FATAL BLEEDING AFTER HAVING SIXTEEN TEETH PULLED.—Reading, Pa. July 9.—Death came swiftly to Miss Hannah Simons as the result of having her teeth extracted. She bled to death, and the coroner is considering the advisability of holding an inquest.

A strange feature of the case is that she said frequently she was afraid to have the teeth extracted, as she thought the operation would kill her. Miss Simons repeated this apprehension as she dropped into Dr. H. L. Johnson's dentist chair Sunday. Miss Simons was employed as a cook in the Hotel Penn cafe. With three girl friends she went to Dentist Johnson's office about noon. She informed the tooth extractor that she had sixteen troublesome teeth, and she wanted them all pulled. Dr. Johnson says he advised Miss Simons to have but eight extracted at one time. He also informed her that it would be necessary to take ether, and Dr. Sterly was summoned to administer the drug.

The sixteen teeth were extracted, and immediately following the operation there was a violent flow of blood. This the dentist seemed unable to stop. Miss Simons was taken to her room and put to bed. The hemorrhage continued, and three hours later Dr. Sterly was called in to see her. He said the flow of blood could not be stopped. So it went on until eight o'clock in the evening, when Dr. Brockbill was summoned. He pronounced Miss Simons to be in a dying condition. The hemorrhage was not checked, and two hours later the girl expired.

In explanation of the fatal results of his operation, Dr. Johnson said that Miss Simons was subject to violent hemorrhages of the nose, and that her gums were in a spongy condition, owing to the decay of her teeth. He said he didn't know she was a victim to nose bleeding until after he had done the operation, or otherwise he would not have extracted the teeth.—*Baltimore Sun.*

---

IRON AND THE TEETH.—New forms of iron, that strong tonic and reconstructive, are introduced from time to time by scientific pharmacists and chemists, but some physicians still stick by their old friend the tincture of the chloride of iron in spite of objections to it. Not only is it very unpleasant to take, but the effects on the teeth are very serious and lasting.

In the *Dominion Dental Journal,* Dr. G. D. Martin, a dentist of Toronto, takes up the effects of this form of iron on the teeth and thinks that it is a very delicate question as affecting the relations existing between physicians and dentists. He says it is clearly laid down in works on materia medica and therapeutics, that this form of iron is especially injurious to to the teeth and should be used with great caution, and yet he is led to believe from his own and others, experience that physicians are lamentably careless and not conscientious in not not warning patients against its dangers.

Druggists have informed him that most of the prescriptions containing this form or any form of iron include no word of caution about the injury that may be caused to the teeth.

He says that it has been shown by experiments on teeth that the tincture of the ferric chloride diluted is even more dangerous and injurious to the teeth than when undiluted. That manufacturing pharmacists are aware of this danger is evidenced by the large number of elegant preparations of iron made with the object of avoiding this dangerous element corroding the teeth. Even the use of alkaline gargles, both before and after taking the iron, may be the best way of counteracting the effects of the iron, but it does not prevent it effectually. Dentists cannot say what remedies physicians shall use, but they can point out to physicians the harm done to the teeth.

The point of the writer is well taken and physicians are not sufficiently careful in prescribing iron, for even if taken through a tube some of the acid will in time affect the teeth if the iron be taken for a long time.—"Md. Med. Journal."

---

CIRCUMCISION.—The nervous system of the infant and of the infant and growing child is so delicately balanced that any irritation if continued is likely to produce vague, er even pronounced, trouble, often at such a great distance from the seat of irritation, that the real cause of the trouble is little suspected except by the most expert. It is partly on this account that Dr. Stone in a late issue advocates circumcision in the case of all infants.

While the feasibility of a wholesale preputial amputation may be questioned, still there are so many cases where the shortening of the covering of the glans penis would be of benefit, that the family physician should examine every infant under his care with the idea of circumcision. That it does cause irritation from the tight constriction of the foreskin, from the collection of smegma and other excretions behind the head of the glans penis, is a fact of every day observation. The Jews who as a part of the old Hebrew ritual, perform this operation on all male children very soon after birth, have been shown to be freer from disorders caused by long prepuce than other races.

The operation, as Dr. Stone Shows, is easy and simple, producing little pain; but care and antiseptic precautions should be used, and if the rites of the Jews demand that the circumciser should perform the operation, he should do it in the most approved manner and not infect the wound of the cut foreskin. Even if vague symptoms referred to a phimosis or paraphimosis do not disappear on completion of the operation, improvement may come later, and even if no improvement result at all the operation will have done no harm.

In the present craze for major operations, such little trivial work is apt to be neglected.

The infant and young child cannot give a clear picture of its trouble and the pediatrist, like the veterinarian, must rely on objective signs and use his powers of observation and his good sense.—"Md. Med. Journal."

---

ALUMINUM PLATES.—I have almost adandoned vulcanite for full sets, and as my failures have taught me more than my successes, I want to explain why so many fail. In the first place, aluminum is adulterated like almost everything else. We might justly call this the age of adulteration. it gets into everything—even into our religion; and we have adulterated clergymen just as we have adulterated dentists. You must get the aluminum free from any adulteration. You can get pure gold if you try, so you can get pure aluminum. In making your models do not put anything whatever in the plaster to harden it, and do not put anything in to harden the the plaster you use in the flasks. Use iron, not brass flasks. I am indebted to Dr. Steele for a hint which has removed my last difficulty, and that was a difficulty which puzzled me often. I used to strike up the aluminum directly between the die metals, and, of course, I got some trace of these metals on the aluminum. I now cover both die and counter die with a thin, tough tissue paper when swaging, using it double immediately over the plate. To make the vulcanite adhere to the aluminum you may either punch holes through it, or with a graver cut in and raise the metal without going through it.—L. D. S. in *Dominion Dental Journal.*

STONED BURS.—It is a comparatively easy matter for the dentist to stone his own burs, whether those previously stoned and worn or the ordinary cavity burs. It requires but a short time to bring each leaf of the bur to an edge with the use of a knife blade-shaped Arkansas stone, examining the bur occasionally during the process under a pocket microscope or hand magnifier. It is essential to perfect work that the stone be kept at a keen edge, and this is easily done as follows: Take a strip of sheet lead three inches long and attach it *evenly* upon a straight and *perfectly* level piece of wood about two-and a-half inches wide and a little longer than the lead. Allow the lead to extend over one end and the sides of the wood, bend the lead over and tack it to the wood over the sides and end—*not on the top*. Upon the top surface thus formed sprinkle some No. 1 emery, and without wetting or oiling the surface rub the stone lengthwise with the strip. Not more than three minutes rubbing of the stone, after it has been used to sharpen a dozen burs, will be necessary to bring it to the required edge again.—*New England Journal of Dentistry.*

---

J. Thomas Lippincott, D. D. S., Demonstrator, Operative Dentistry, University of Pennsylvania. writes: "I can cheerfully recommend Borine as one of the most pleasant and efficient aids in the treatment of inflamed conditions of the gums and mucous membrane."

The fact that decay of the teeth is of parasitic origin having been once established the thought suggests itself that it should be possible by means of properly chosen antiseptic materials, not only to arrest decay but to prevent its appearance. This is actually what Borine does and will accomplish.

Borine will devitalize the mouth in ¼ minute, while it does not harm the delicate Epithelium, nor injure the dentine as nearly all other antiseptics do.

Borine is of absolute necessity to wearers of artificial dentures.

CHARACTER IN TEETH.—Character reading from handwriting, from shoes and from the face, has now been succeeded by character-reading from the teeth. A dentist explains in a contemporary that a careful study of teeth will reveal the fact that they invariably indicate, according to their shape and setting, the temperament of their possessors. One has only to note the teeth of one's friends and relations to verify his observations on pointed, projecting, short, square, tangled, even and pearly dentures. Those that are long and narrow, we are assured, denote vanity; those that are long and projecting indicate a grasping disposition; treachery is shown by the possession of small, white separated teeth; and inconstancy is revealed by overlapping teeth.—*Lady's Pictorial.*

---

BEESWAX IN CANALS.—Dr. Ives uses beeswax for filling root canals, having discarded all other materials for it. The beeswax is rolled to a point; inserted in canal, melted into tubuli by use of an Evans root drier, more wax being added until canal is full. A copper point is then heated and sent right to the end, the distance having first been measured. He thinks that in this way every part of the root is filled, and as beeswax does not shrink or expand, and is not affected by acids or alkalies, he is confident that it is the best of all materials within our reach.—*Dominion Dental Journal.*

---

POISONING BY LYSOL.—This antiseptic has caused the death of a child to whom it was administered by mistake instead of a laxative. Dr. Haberda has shown that the poisonous action of lysol is due to its containing kresols. It cauterizes the skin and mucous membrane, and when absorbed it affects the brain and spinal cord, producing unconsciousness, general spasms, reduction of temperature, and bleeding into the uriniferous tubules. He therefore recommends that it should be prescribed only in dilute solution.—*British Journ. Dental Science.*

RULES FOR THE SETTING OF OXY-PHOSPHATE CEMENT FILLINGS.—If the cement sets too quickly add a few drops of saturated solution of borax and water to the bottle of liquid to make it set slower.

If the cement sets too slowly add a few drops of pure hydrochloric acid to the bottle of liquid to make it set quicker.

If your bottle of liquid be full four drops of either will suffice.

If your bottle of liquid be two-thirds full three drops may be added, and if only half full two drops will be sufficient. Whatever be the quantity of liquid in your bottle, the borax solution or the hydrochloric acid may be added in these proportions.—*Microcosm.*

---

A COMPATIBLE ANTISEPTIC.—Dr. Baxter, of this city, in referring to antiseptics, thus commends the compound Listerine: "The genial compatibility of Listerine with so many standard remedies of the materia medica gives it a very wide range of applicability in the treatment of that large class of cases benefitted, relieved by the antiseptic treatment. It has served me well in gonorrhœ, catarrh, fistula in ano, and offensive discharges from the ear and uterus. It is the most elegant mouth wash I have ever used, and for dental use must prove invaluable."—"Dominion Dental Journal."

---

TO LESSEN THE PAIN FROM ARSENIC.—Mix equal parts antipyrin and arsenic. The antipyrin diminishes blood-pressure and relieves the congestion caused by the arsenic, and therefore diminishes pain.—G. C. Richards, in *Microcosm.*

